# Enhancer Sharing Promotes Neighborhoods of Transcriptional Regulation Across Eukaryotes

**DOI:** 10.1534/g3.116.036228

**Published:** 2016-10-31

**Authors:** Porfirio Quintero-Cadena, Paul W. Sternberg

**Affiliations:** Division of Biology and Biological Engineering, California Institute of Technology, Howard Hughes Medical Institute, Pasadena, California 91125

**Keywords:** enhancer sharing, gene coexpression, gene neighbors, enhancer specificity, transcriptional domains

## Abstract

Enhancers physically interact with transcriptional promoters, looping over distances that can span multiple regulatory elements. Given that enhancer–promoter (EP) interactions generally occur via common protein complexes, it is unclear whether EP pairing is predominantly deterministic or proximity guided. Here, we present cross-organismic evidence suggesting that most EP pairs are compatible, largely determined by physical proximity rather than specific interactions. By reanalyzing transcriptome datasets, we find that the transcription of gene neighbors is correlated over distances that scale with genome size. We experimentally show that nonspecific EP interactions can explain such correlation, and that EP distance acts as a scaling factor for the transcriptional influence of an enhancer. We propose that enhancer sharing is commonplace among eukaryotes, and that EP distance is an important layer of information in gene regulation.

Enhancers mediate the transcriptional regulation of gene expression, enabling isogenic cells to exhibit remarkable phenotypic diversity (Davidson and Peter 2015). In complex with transcription factors, they interact with promoters via chromatin looping (Marsman and Horsfield 2012), finely regulating transcription in time and space. A prevailing view is that most enhancers have a mechanism to selectively loop to a target promoter (van Arensbergen *et al.* 2014). Examples in this category usually require specific transcription factor binding at both enhancer and promoter sites (Davidson and Peter 2015), which could explain why some enhancers seem to influence different promoters to varying degrees (Gehrig *et al.* 2009). On the other hand, EP looping is generally mediated by common protein complexes (Kagey *et al.* 2010; Malik and Roeder 2010), conflicting with the specific molecular interactions required by such a model at a larger scale. Examples of nonspecific EP pairing also seem to be common (Butler and Kadonaga 2001). Yet given that this model could result in transcriptional crosstalk, it appears inconsistent with our current paradigm of gene regulation. The predominant EP pairing scheme, specific or nonspecific, and its determinants are thus unclear. Here, we ask to what extent are potential EP pairs compatible through a meta-analysis of the genome-wide transcription of gene neighbors in five species. We propose that enhancer sharing occurs widely across eukaryotes, test key aspects of this hypothesis in *Caenorhabditis elegans*, and analyze its implications in other genomic phenomena.

## Materials and Methods

### Computational biology

For each analyzed organism, Ensembl (Flicek *et al.* 2014) protein-coding genes were grouped by chromosome, sorted by position, and paired with the 100 nearest neighbors within the same chromosome. A list of duplicated gene pairs for *Homo sapiens* and *Mus musculus* was obtained from the Duplicated Genes Database (Ouedraogo *et al.* 2012) (http://dgd.genouest.org). A list of *C. elegans* genes predicted to be in operons was obtained from Allen *et al.* (2011), and gene pairs present in the same operon were removed from the analysis to prevent cotranscriptional bias. Processed RNA-seq data were obtained from multiple sources (Gerstein *et al.* 2010; Attrill *et al.* 2016; Ellahi *et al.* 2015; The ENCODE Project Consortium 2012) and converted to transcripts per million (TPM) (Li *et al.* 2010) when necessary. Formatted datasets are available upon request. Genes detected in < 80% of experiments were discarded. To compute the Spearman correlation coefficient, TPM values were ranked in each RNA-seq experiment and the pairwise Pearson correlation coefficient was computed on the ranked values according to the following equation:

$$\rho =\frac{\mathrm{cov}\left({\text{gene}}_{1},{\text{gene}}_{2}\right)}{\sigma_{{\text{gene}}_{1}}\sigma_{{\text{gene}}_{2}}}$$

where ${\text{gene}}_{1}$ and ${\text{gene}}_{2}$ are the corresponding ranks of each paired gene in a given RNA-seq experiment, cov their covariance and σ their SD. The list of gene pairs with intergenic distances and correlation coefficients was sorted by increasing intergenic distance, and subsequently smoothed using a sliding median with window size of 1000 gene pairs. The result was then fitted to an exponential decay function of the form:

$$\rho \left(d\right)=\rho_{0}e^{-\lambda d}+c$$

where $\rho_{0}$ is the median Spearman correlation coefficient of the closest neighboring genes, *d* the intergenic distance, and *c* the baseline correlation. The mean distance at which a pair of genes remain correlated was then computed as:

$$d_{exp}=1/\lambda$$

To compute the background correlation, each gene was paired with 20 randomly selected genes from a different chromosome and the 95% median confidence interval was computed by bootstrapping with 10,000 samples. A list of genes annotated with RNA *in situ* hybridization data (Hammonds *et al.* 2013; Tomancak *et al.* 2002, 2007) was obtained from the Berkeley *Drosophila* Genome Project (http://insitu.fruitfly.org). Insulator Chromatin ImmunoPrecipitation coupled with microarrays (ChIP-chip) data were obtained from Negre *et al.* (2010) (GSE16245); the intersection of replicates was used. HiC data were obtained from Rao *et al.* (2014) (GSE63525, GM12878 primary replicate HiCCUPS looplist). Functional protein classification was conducted using Panther (Mi *et al.* 2016). Genomic manipulations were conducted using Bedtools v2.24.0 (Quinlan and Hall 2010). Data analysis was conducted using Python 2.7.9 and the Scipy library (McKinney 2010). Plots were generated using Matplotlib 1.5 (Hunter 2007).

### Molecular biology

*C. elegans* was cultured under standard laboratory conditions (Stiernagle 2006). For enhancer additivity experiments, transgenic *C. elegans* lines carrying extrachromosomal arrays were generated by injecting each plasmid at 50 ng/μl into *unc-119* mutant animals. The minimal Δ*pes-10* promoter (Fire *et al.* 1990) and nuclear localized GFP (Lyssenko *et al.* 2007) were used in all constructs. Minimal regions of the *myo-2* and *unc-54* enhancers (Okkema *et al.* 1993) able to drive tissue-specific expression were used. The BWM (body wall muscle) enhancer was obtained from the upstream region of *F44B9.2*; the BWM/intestine enhancer was obtained from the upstream region of *rps-1*. Animals were imaged at 40 × using a GFP filter on a Zeiss Axioskop microscope.

For the EP distance and ectopic enhancer experiments, we defined an EP distance of 0 to be the enhancer placed just upstream of the Δ*pes-10* promoter, which is ∼350 bp away from the start codon of *gfp*. To ensure neutrality yet maintain a similar GC content as noncoding sequences in *C. elegans*, we used nonoverlapping AT-rich DNA spacers obtained from the genome of *Escherichia coli*. Constructs were integrated in single-copy into chromosome IV via CRISPR-Cas9 using plasmids provided as gifts by Zhiping Wang and Yishi Jin (unpublished results). Briefly, plasmids containing the following expression cassettes were coinjected: reporter and hygromycin resistance genes flanked by homologous arms for recombination-directed repair (10 ng/μl), single-guide RNA (10 ng/μl), Cas9 (10 ng/μl), and an extrachromosomal array reporter for expression of either *rfp* or *gfp* outside the tissue of interest (10 ng/μl). Transformants were selected for using hygromycin at 10 μg/μl, and those not carrying extrachromosomal transgenes, which lacked *gfp* or *rfp* expression outside the tissue of interest, were subsequently isolated. Animals homozygotic for the insertion were identified by polymerase chain reaction (PCR) and Sanger sequencing.

Quantitative PCR was carried out as previously described (Ly *et al.* 2015) using *pmp-3* as a reference gene (Zhang *et al.* 2012). Briefly, third-stage larval (L3) worms, when expression from the test enhancers is maximal according to RNA-seq data, were synchronized at 20° via egg-laying. Fifteen animals were lysed in 1.5 μl of Lysis Buffer [5 mM Tris pH 8.0 (MP Biomedicals), 0.5% Triton X-100, 0.5% Tween 20, 0.25 mM EDTA (Sigma-Aldrich)] with proteinase-K (Roche) at 1.5 μg/μl, and incubated at 65° for 10 min followed by 85° for 1 min. Reverse transcription was carried out using the Maxima H Minus cDNA synthesis kit (Thermo Fisher Scientific) by adding 0.3 μl H_2_O, 0.6 μl 5 × enzyme buffer, 0.15 μl 10 mM dNTP mix, 0.15 μl 0.5 μg/μl oligo dT primer, 0.15 μl enzyme mix, and 0.15 μl DNAse, and incubated for 2 min at 37°, followed by 30 min at 50°, and finally 2 min at 85°. The cDNA solution was diluted to 15 μl and 1 μl was used for each qPCR reaction, so that on average each well contained the amount of RNA from a single worm. All qPCR reactions were performed with three technical replicates and at least three biological replicates using the Roche LightCycler 480 SYBR Green I Master in the LightCycler 480 System. Crossing point-PCR-cycle (Cp) averages were computed for each group of three technical replicates; these values were then subtracted from the respective average Cp value of the reference gene.

### Data and reagent availability

Strains are available upon request. Relevant DNA sequences, including spacers, enhancers, primers, sgRNA, and homology arms are available in Supplemental Material, Table S1. Correlation datasets are available in File S1 and File S2. qPCR data are available in Table S2. Python source code, and links to all expression datasets used in this study, are available for download on the following github repository: https://github.com/WormLabCaltech/QuinteroSternberg2016.git.

## Results and Discussion

### Gene neighbors are transcriptionally correlated genome-wide

We reasoned that widespread EP compatibility should result in transcriptional correlation among gene neighbors. Indeed, gene coexpression clusters have been extensively reported in eukaryotic genomes (*e.g.*, Sémon and Duret 2006; Roy *et al.* 2002; Lercher *et al.* 2002, 2003; Lercher and Hurst 2006; Williams and Hurst 2002; Singer *et al.* 2005; Williams and Bowles 2004; Spellman and Rubin 2002; Purmann *et al.* 2007; Zhan *et al.* 2006; Boutanae v *et al.* 2002; Kalmykova *et al.* 2005; Caron *et al.* 2001; Rubin and Green 2013) in spite of order of magnitude variations in genome size (*e.g.*, ∼12 Mb in *Saccharomyces cerevisiae vs.* ∼3 Gb in *H. sapiens*). An early informative example is the discovery of chromosomal domains of gene expression in *S. cerevisiae* (Cohen *et al.* 2000) that exhibit features that strongly support enhancer-sharing, mainly distance-dependence in transcriptional correlation that qualitatively resemble chromosome contact matrices (*e.g.*, Rao *et al.* 2014), and instances in which a single enhancer seems to be responsible for the coexpression of adjacent gene pairs. The ubiquity of these features across eukaryotes would support the idea that EP interactions are largely determined by physical proximity rather than by specific interactions. Given the accumulation of transcriptome sequencing data, we decided to investigate the transcriptional correlation of gene neighbors in representative eukaryotic species as a first step to explore the average EP pairing scheme.

We paired every protein-coding gene of five organisms (*S. cerevisiae*, *C. elegans*, *Drosophila melanogaster*, *M. musculus*, and *H. sapiens*) with its 100 nearest neighbors within the same chromosome. This yielded lists of around 600,000 (*S. cerevisiae*) and 2 million (each of the rest) gene pairs. We then computed the Spearman correlation coefficient between paired genes across multiple RNA-seq datasets (Gerstein *et al.* 2010; Attrill *et al.* 2016; Ellahi *et al.* 2015; The ENCODE Project Consortium 2012) and the intergenic distance between the start of the 5′ untranslated region of the first gene to the start of the second gene in each pair.

**Figure 1:**
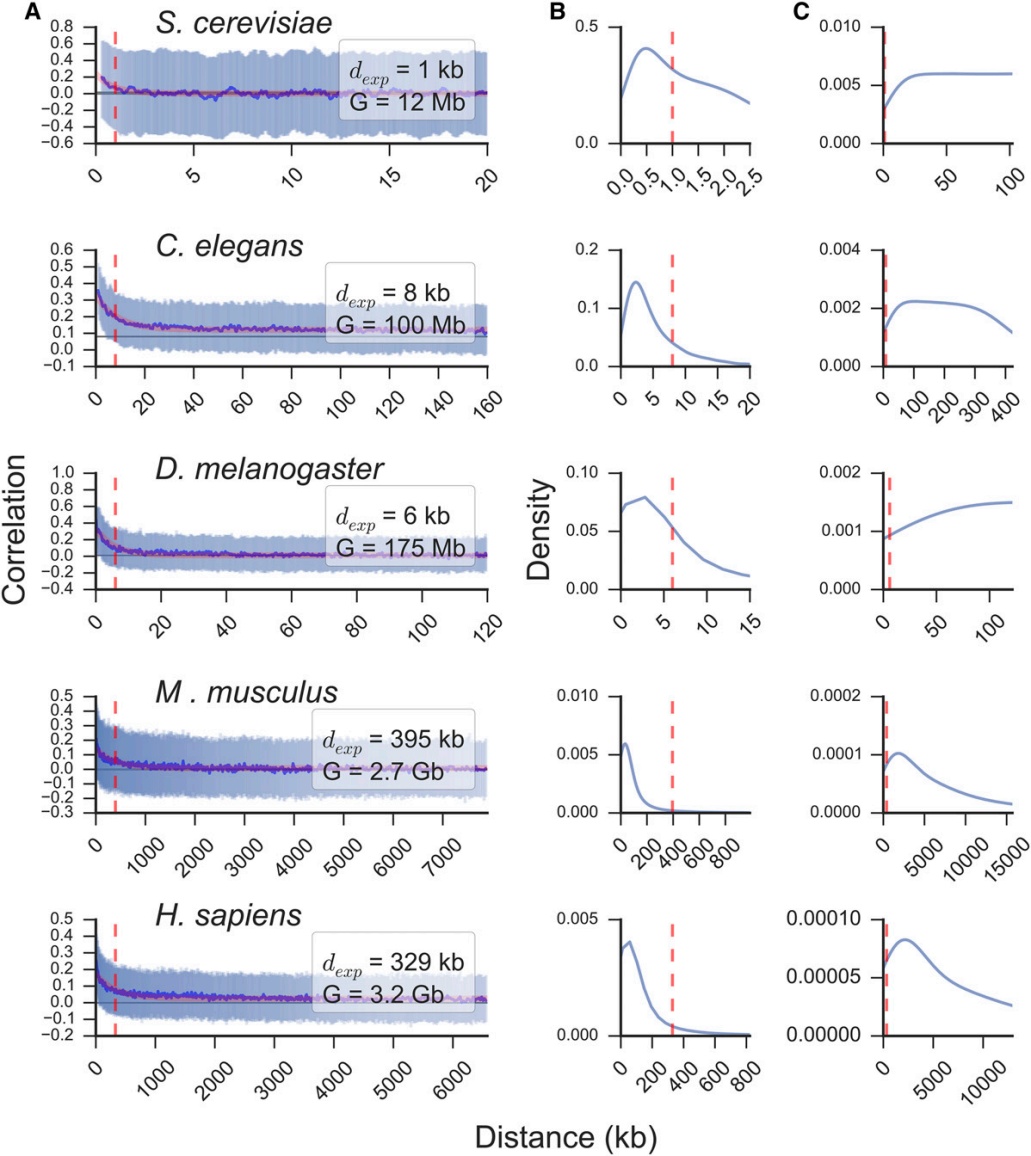
Neighboring genes are transcriptionally correlated genome-wide across eukaryotes. (A) Sliding median of correlations between paired neighbors (blue line) and interquartile range (pale blue) with increasing intergenic distance. Median ± 95% C.I. of randomly paired genes is shown as a horizontal gray line. Fit to an exponential decay function (red line) was used to compute the mean distance at which gene neighbors remain correlated ($d_{\exp},$ vertical red dashed line). The genome size (G) is displayed for each organism. Distribution of intergenic distances between each gene and its nearest neighbor (B) and all paired genes (C). The organism analyzed in each case is indicated for each group of three subplots.

We observed that neighboring genes tend to be correlated in transcript abundance genome-wide in all analyzed organisms, and that this correlation decays exponentially with increasing intergenic distance (Figure 1A). We thus fitted the data to an exponential decay function to estimate the distance at which a pair of genes remain correlated ($d_{\exp}$). Consistent with the persistence of the correlation pattern across organisms, $d_{\exp}$ scaled with genome size, to 1 kb in *S. cerevisiae*, ∼10 kb in *C. elegans* and *D. melanogaster*, and ∼350 kb in *M. musculus* and *H. sapiens* (Figure S1). This trend remained largely the same even after removing duplicated gene pairs (Figure S2). Most genes had at least one neighbor closer than $d_{\exp}$ in all species (Figure 1B), and the representation of gene ontology annotations remained unbiased in correlated gene pairs (Figure S3), indicating that the average gene is correlated in expression with its nearest neighbors beyond any particular gene class. In addition, sampled intergenic distances go well beyond $d_{\exp}$ (Figure 1C), indicating that 100 gene neighbors are a sufficient number to study this effect.

To examine the correlation of gene expression in the spatial domain, we analyzed RNA *in situ* hybridization data for 6053 genes in *D. melanogaster* (Tomancak *et al.* 2002, 2007; Hammonds *et al.* 2013). We computed the percentage overlap in tissue expression by dividing the number of common tissues over the total number of unique tissues in which genes of any given pair are expressed (Figure S4A). This analysis revealed that close neighbors have a tendency to be expressed in the same tissues, and that this overlap also decays exponentially with intergenic distance (Figure S4B). However, the correlation extends to a longer mean distance ($d_{\exp}$ = 22 compared to 6 kb), suggesting that RNA-seq analysis, which included mostly whole-organism transcriptome averages, resulted in a conservative estimate.

Given that pairing every gene with 100 proximal genes provides a complete set of distance-dependent correlations between gene pairs, we concluded that gene neighbors have a spatio-temporal correlation in expression that is highly dependent upon the spacing between them. Our meta-analysis unifies the findings of previous reports (reviewed in Michalak 2008) and highlights the distance-dependence of genome-wide and cross-organismic transcriptional correlations that transcend localized gene coexpression clusters.

### Enhancer sharing explains the transcriptional correlation of gene neighbors

**Figure 2:**
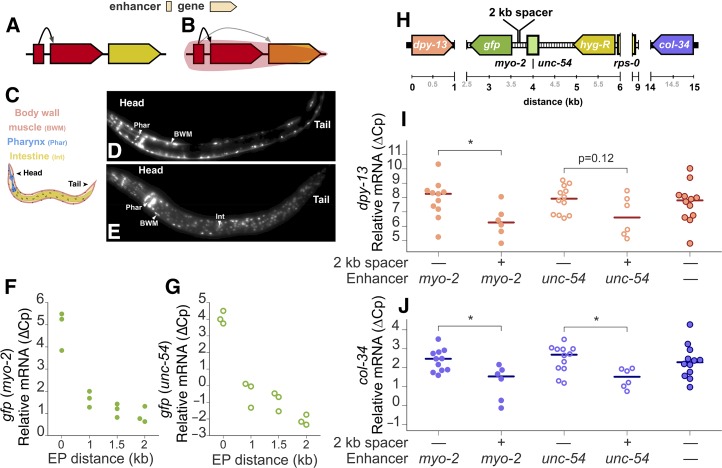
Enhancer sharing explains the transcriptional correlation of gene neighbors. Two possible models for EP relationship: (A) Enhancers have specific target genes and (B) enhancers have a range of action in which they influence genes by physical proximity. Tissue-specific enhancers (C) are generally compatible. Pharynx and body wall muscle (D) and pharynx, body wall muscle, and intestine (E) enhancers driving nuclear *gfp* expression. mRNA levels of *gfp* with increasing EP distance for lines with *myo-2* (filled circles) (F) and *unc-54* (hollow circles) (G) enhancers. (H) Genomic context of the integration site. The inserted construct is shown over a dashed black line and includes a hygromycin resistance gene (*hyg-R*) regulated by a ribosomal enhancer (*rps-0*) and promoter in addition to the reporter (*gfp*) regulated by either the *myo-2* or *unc-54* enhancers; the native genes *dpy-13* and *col-34* flank the insertion site. Relative mRNA levels of *dpy-13* (I) and *col-34* (J) in wild-type and lines with and without the 2 kb spacer (* two tailed *P*-value < 0.05, Mann-Whitney *U*-test). The difference in crossing point-PCR-cycle (ΔCp) with the reference gene *pmp-3* and the corresponding median for each group of biological replicates is shown for every qPCR experiment. EP, enhancer–promoter; mRNA, messenger RNA; PCR, polymerase chain reaction; qPCR, quantitative PCR.

The pervasive nature of proximal gene coexpression supported the idea of widespread EP compatibility. This connection is, in turn, supported by several other observations in the literature: (i) enhancers regulate transcription by making contact with promoters via chromatin looping (Marsman and Horsfield 2012), whose incidence also decays exponentially as the distance between contacting sites increases (Ringrose *et al.* 1999; Rao *et al.* 2014), with the same pattern as observed here at least in some documented cases (*e.g.*, *H. sapiens*, Figure S5); (ii) the average distance between a large fraction of studied EP interactions scales with genome size in ranges often consistent with $d_{\exp}$ < 1 kb in *S. cerevisiae*, (Dobi and Winston 2007), < 10 kb in *C. elegans*, (Araya *et al.* 2014), and 120 kb in *H. sapiens* (Sanyal *et al.* 2012); (iii) common protein complexes such as the mediator seem to be widely utilized bridges in EP looping (Kagey *et al.* 2010; Malik and Roeder 2010); (iv) a high frequency of chromatin interactions are observed within topologically-associated domains identified through high-resolution Chromosome Conformation Capture (Hi-C) (Rao *et al.* 2014); and (v) studied enhancers often do not show promoter specificity (Butler and Kadonaga 2001). This line of reasoning suggests a model where, as opposed to only having a specific target gene (Figure 2A), the average enhancer has a range of action in which it can influence any active promoter within its reach (Figure 2B). A concrete example consistent with this idea is the upregulation of neighboring genes upon enhancer activation by fibroblast growth factor in mammalian cells (Ebisuya *et al.* 2008). Transcriptome analysis could thus provide indirect evidence of genome and condition-wide EP looping that is difficult to access through Hi-C (Rao *et al.* 2014) due to the low signal-to-noise ratio of short-range interactions.

Because of its compact genome, rapid development, and availability of tissue-specific enhancers (Corsi *et al.* 2015), we decided to use *C. elegans* to test the validity of a nonspecific EP pairing model. We first postulated that unrelated enhancers should generally be compatible, showing qualitative additivity when placed upstream of a single promoter. We thus paired the well-characterized *myo-2* pharyngeal enhancer with a BWM and a BWM plus intestine enhancer, placed them upstream of a minimal promoter and a *gfp* reporter, and examined expression in transgenic animals. In both cases, we observed fluorescence in the corresponding tissues (Figure 2, C–E). This observation is consistent with typical enhancer studies in artificial constructs (Dupuy *et al.* 2004) and agrees with some EP compatibility studies (Butler and Kadonaga 2001).

Given that both chromatin looping and expression correlation decay exponentially, we reasoned that transcription of a given gene should also decay exponentially with increasing EP distance if the observed correlation is to be explained by enhancer sharing. To test this hypothesis, we first built a series of genetic constructs with increasing neutral EP distances (0, 1, 1.5, and 2 kb) for two different enhancers, *myo-2* and *unc-54* (∼400 and 300 bp, respectively). We then integrated each construct in single-copy into the genome of *C. elegans* and used quantitative PCR to: (i) measure the influence of EP distance on the reporter gene in native chromatin and (ii) analyze the impact of the perturbation on the two genes that natively flank the site of transgene insertion (*dpy-13* and *col-34*, Figure 2H), which we reasoned should be affected in two counteracting ways. First, the ectopic enhancers should promote their expression. Second, the increased EP distance caused by the addition of spacers should reduce their expression by scaling down the influence of both ectopic and native enhancers (the latter of unknown identity and location) to the opposite side of the spacer.

We found that transcriptional levels of the reporter gene indeed fall rapidly with increasing EP distance with both enhancers (Figure 2, F and G); this occurred at a rate that seems congruent or faster than the genome-wide correlation decay, likely because of the dramatic separation of every regulatory element at once, as opposed to gradual separation from individual enhancers in a native environment; this dramatic effect suggests complex interactions between multiple EP loops that are disrupted with the insertion of DNA sequences devoid of regulatory elements. Transcription was still well detected even when the enhancers were placed 2 kb away, supporting the hypothesis that EP distance is a scaling factor on the enhancer’s influence. Expression of *dpy-13* and *col-34* was reduced with the introduction of the 2 kb spacer when compared to transgenic lines without it (Figure 2, I and J). On the other hand, spacer-free lines were comparable to wild-type, suggesting that the incorporation of ectopic enhancers compensated for the EP distance increase caused by the addition of the genetic construct itself. These observations seem to fit the corollaries of our model, even amid the complexity of a native regulatory environment. However, the distance over which we see an effect on *col-34* falls outside our $d_{\exp}$ estimate for *C. elegans* (8 kb). Its expression is impacted by the presence of the 2 kb spacer outside of the interval between the *myo-2*/*unc-54* enhancer, suggesting that enhancers >12 kb away can still influence its expression. As evidenced with the discrepancy in *D. melanogaster* when using *in situ* or RNA-seq data, this observation suggests that $d_{\exp}$ is only a rough estimate of the average enhancer rage of action; this is useful to gain insight into genome-wide mechanisms but not for precise individual predictions.

Chromatin modifications have been shown to have a significant impact on enhancer function (Calo and Wysocka 2013) and thus likely influence EP pairing. Thus, chromatin features and enhancer sharing might be mutually inclusive rather than stand-alone explanations for the observed correlation domains. From this perspective, transcriptionally correlated genes would have similar chromatin states, facilitated by their physical proximity, that make them accessible to enhancer action.

The existence of multiple, independent, but similar enhancers is an alternative possible explanation. However, since we are looking at genome-wide averages, this would mean that most gene neighbors have a functionally redundant set of independent enhancers that function through distinct molecular interactions. Although possible, this is a rather intricate explanation.

In agreement with the enhancer sharing hypothesis, it can be argued that the scaling of correlation domains is a result of the ability to connect EP loops over longer distances in larger genomes. Yet, in spite of having a compact genome, *D. melanogaster* is able to form many long-range EP interactions (>50 kb) (Ghavi-Helm *et al.* 2014), which is considerably different to the range of its estimated $d_{\exp}$ (6–22 kb). Additionally, these long-range interactions appear to be particularly specific, with enhancers selectively activating their target promoters (Ghavi-Helm *et al.* 2014; Kwon *et al.* 2009). It is, thus, possible that in compact genomes, long-range EP interactions would need to be specific, whereas nearby interactions would tend to fall in the nonspecific pairing scheme, ultimately resulting in the observed correlation domain size.

### EP distance insulates neighboring genes

**Figure 3:**
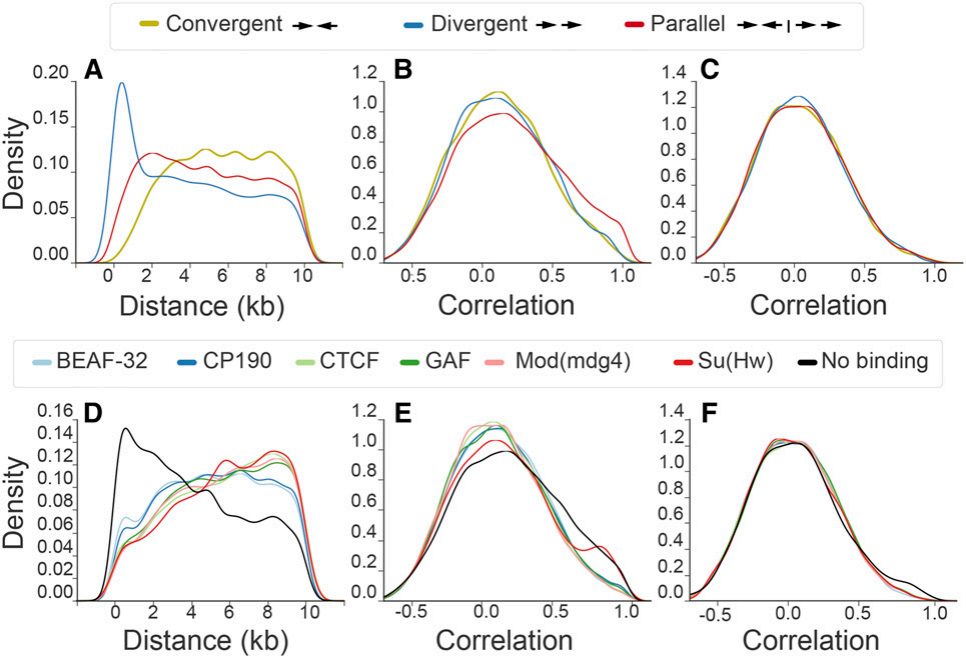
EP distance causes gene orientation-dependent correlation and provides regulatory independence to gene neighbors. Distribution of intergenic distances < 10 kb of gene pairs in *D. melanogaster* by configuration (∼5–18,000 gene pairs for each group) (A) and flanking insulator binding sites identified through ChIP-chip (Negre *et al.* 2010) (∼5–15,000 pairs for each group) (D). The corresponding distribution of correlations is shown for the same gene pairs (B, E) and pairs with controlled distributions of intergenic distances between 30 and 40 kb (∼7–14,000 pairs for gene orientation groups, ∼10–18,000 for insulator groups) (C and F). CHIp-chip, Chromatin ImmunoPrecipitation coupled with microarrays; EP, enhancer–promoter.

We next wished to determine the extent to which enhancer sharing could explain other genomic phenomena. Previous reports have suggested that divergent, parallel, and convergent gene pairs tend to have distinct correlation profiles (*e.g.*, Chen and Stein 2006). To explore this hypothesis, we compared the distribution of intergenic distances of gene pairs oriented in parallel, divergent, and convergent orientations (Figure 3A and Figure S6). As expected, divergent gene pairs tend to be closest, followed by parallel, and finally convergent genes. We then confirmed that each group appears to have different distributions of correlations (Figure 3B and Figure S6). To consider the influence of EP distance, we sampled gene pairs from each orientation controlling for intergenic size. This resulted in distributions of correlations that exactly overlap (Figure 3C and Figure S6), an observation that is supported by previous reports in specific cases (Ghanbarian and Hurst 2015; Cohen *et al.* 2000). We thus conclude that the apparent influence of gene orientation in the transcriptional relationship of neighboring gene pairs is consistent with the enhancer sharing hypothesis. In this scenario, the effect of gene orientation can be simply explained by the different EP distance distributions for each configuration.

From the regulatory perspective, EP distance provides independence to most gene pairs, as the vast majority have an intergenic distance that puts them in the baseline correlation regime (Figure 1C). To study the enhancer-blocking influence of insulators (Bushey *et al.* 2009) genome-wide, we analyzed each group of genes flanked by insulator binding sites, which were previously determined using ChIP-chip for six known insulators in *D. melanogaster*: BEAF-32, CP190, CTCF, GAF, Mod(mdg4), and Su(Hw) (Negre *et al.* 2010). We observed that gene neighbors closer than 10 kb bound by each of the insulators tend to be less correlated in gene expression than gene pairs not bound by them (Figure 3E), supporting their role in genome-wide insulation and agreeing with the observation that insulators tend to separate differentially expressed genes (Negre *et al.* 2010; Xie *et al.* 2007). Nevertheless, the same groups of gene pairs also tend to have much larger intergenic distances than genes that are not flanked by insulator binding sites (Figure 3D). After controlling for the distribution of intergenic distances, we found very similar correlation distributions between insulator and not insulator flanked gene pairs (Figure 3F). This finding agrees with previous reports suggesting that insulators do not block enhancers everywhere they bind, but rather act only on very specific genomic contexts (Schwartz *et al.* 2012; Liu *et al.* 2015; Ong and Corces 2014); it also reconciles the lack of known insulator orthologs in *C. elegans* (Heger *et al.* 2009) in the context of local enhancer-blocking. In combination, these studies strongly suggest that EP distance is the general source of transcriptional independence for close gene neighbors.

Previous EP compatibility studies have suggested that EP specificity is widespread (Gehrig *et al.* 2009), while others have suggested that it is restricted to a smaller subset of enhancers (Butler and Kadonaga 2001). Although our results support the latter, views arising from these studies might not be mutually exclusive, as it is likely that enhancers have specificity to promoter classes (Danino *et al.* 2015), whose limited number could result in general EP compatibility.

The implications from considering our observations are broadly applicable to gene regulation. Position effects, in which transgene expression levels are influenced by the insertion site (Gierman *et al.* 2007), are naturally expected from enhancer sharing. Chromosomal translocations and mutations involving regulatory elements likely impact genetic contexts rather than individual genes. Furthermore, enhancer sharing and distance-based scaling of enhancer influence potentially provides an additional layer of information in gene regulation, as the transcriptional output of a given gene would be the result of scaled contributions from multiple shared enhancers. Such a feature could, by itself, be under selective pressure, leading to a roughly constant size of the correlation domain in number of genes regardless of absolute physical distance, as observed in this study. Our analysis provides a clarifying perspective of gene regulation consistent with both mechanistic and genome-wide studies.

## Supplementary Material

Supplemental Material
