# Supplementary material for: Enhancer Sharing Promotes Neighborhoods of Transcriptional Regulation Across Eukaryotes
Source: G3 (Bethesda). 2016 Oct 31;6(12):4167–74. doi: 10.1534/g3.116.036228 (PMC5144984; doi:10.1534/g3.116.036228)
Supplement: Supplemental Material [file supp_6_12_4167__index.html]

Enhancer Sharing Promotes Neighborhoods of Transcriptional Regulation Across Eukaryotes — Supplemental Material 

# Enhancer Sharing Promotes Neighborhoods of Transcriptional Regulation Across Eukaryotes

## Supplemental Material for Quintero-Cadena and Sternberg, 2016

**Files in this Data Supplement:**

- File S2 - This file contains all supplemental figures and captions. (.pdf, 302 KB)
- File S1 - Correlation datasets. (.zip, 531.34 MB)
- Figure S1 - The distance at which a pair of genes remain correlated (*dexp*) scales with genome size. (.eps, 121 KB)
- Figure S2 - Removing duplicated genes does not affect the overall correlation of gene neighbors. (.jpg, 4.17 MB)
- Figure S3 - Representation of gene ontology annotations remains unbiased in correlated gene pairs. (.eps, 190 KB)
- Figure S4 - Gene pairs are correlated in spatial expression in *D. melanogaster*. (.jpg, 4.43 MB)
- Figure S5 - Chromatin looping decreases exponentially with distance in human cell lines. (.jpg, 2.13 MB)
- Figure S6 - Gene orientation effect in correlation of gene pairs is explained by EP distance. (.eps, 135 KB)
- Table S1 - Relevant DNA sequences. (.csv, 11 KB)
- Table S2 - qPCR data. (.csv, 9 KB)
