# Supplementary material for: Enhancer Sharing Promotes Neighborhoods of Transcriptional Regulation Across Eukaryotes
Source: G3 (Bethesda). 2016 Oct 31;6(12):4167–74. doi: 10.1534/g3.116.036228 (PMC5144984; doi:10.1534/g3.116.036228)
Supplement: Supplemental Material [file supp_g3.116.036228_FileS2.pdf]

Supplementary figures: Enhancer sharing promotes neighborhoods of transcriptional regulation across eukaryotes (2016)

Porfirio Quintero-Cadena and Paul W. Sternberg

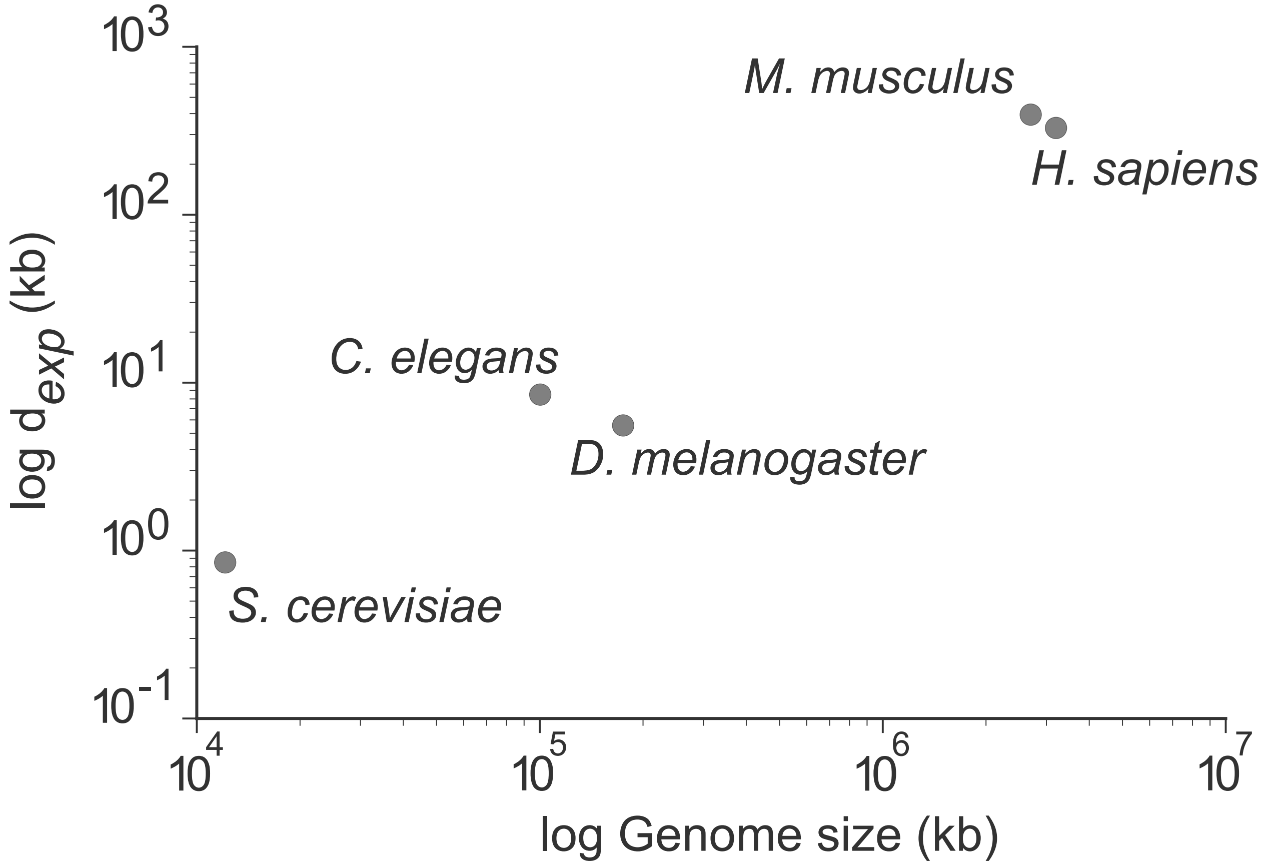

Figure S1 The distance at which a pair of genes remain correlated (d_{exp}) scales with genome size.

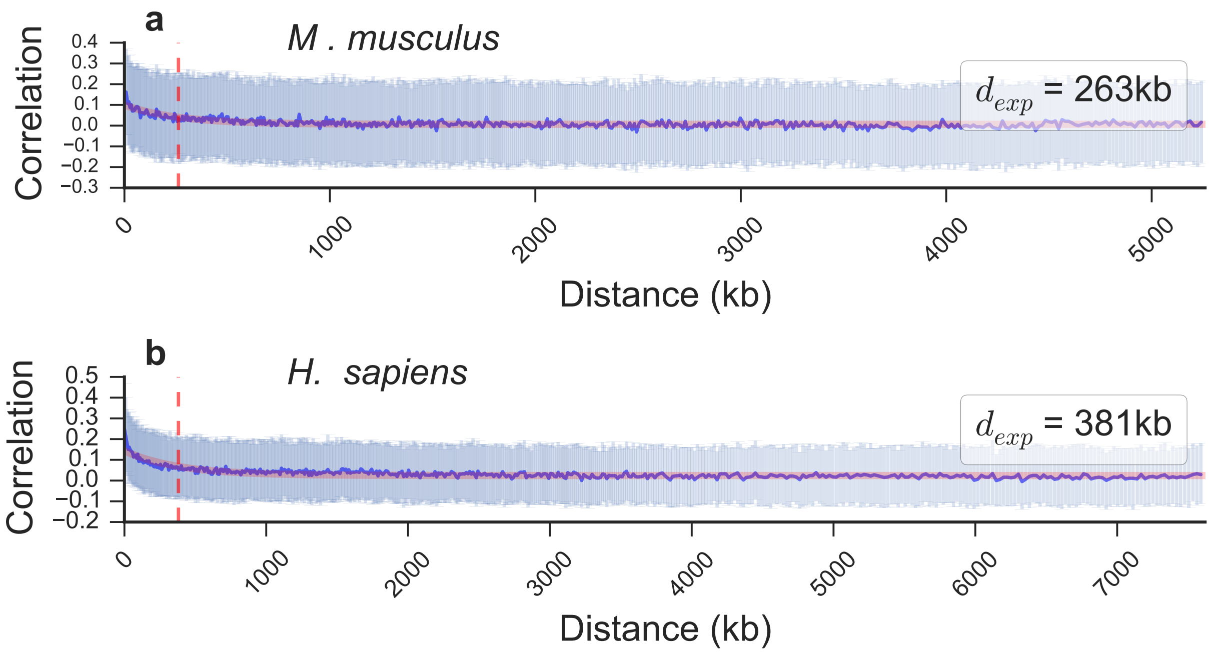

Figure S2 Removing duplicated genes does not affect the overall correlation of gene neighbors. Sliding median of correlations between paired neighbors (blue line) and interquartile range (pale blue) with increasing intergenic distance in *M. musculus* (a) and *H. sapiens* (b) after removing duplicated gene pairs. Fit to exponential decay function (red line) and corresponding d_{exp} (red dashed line) are shown.

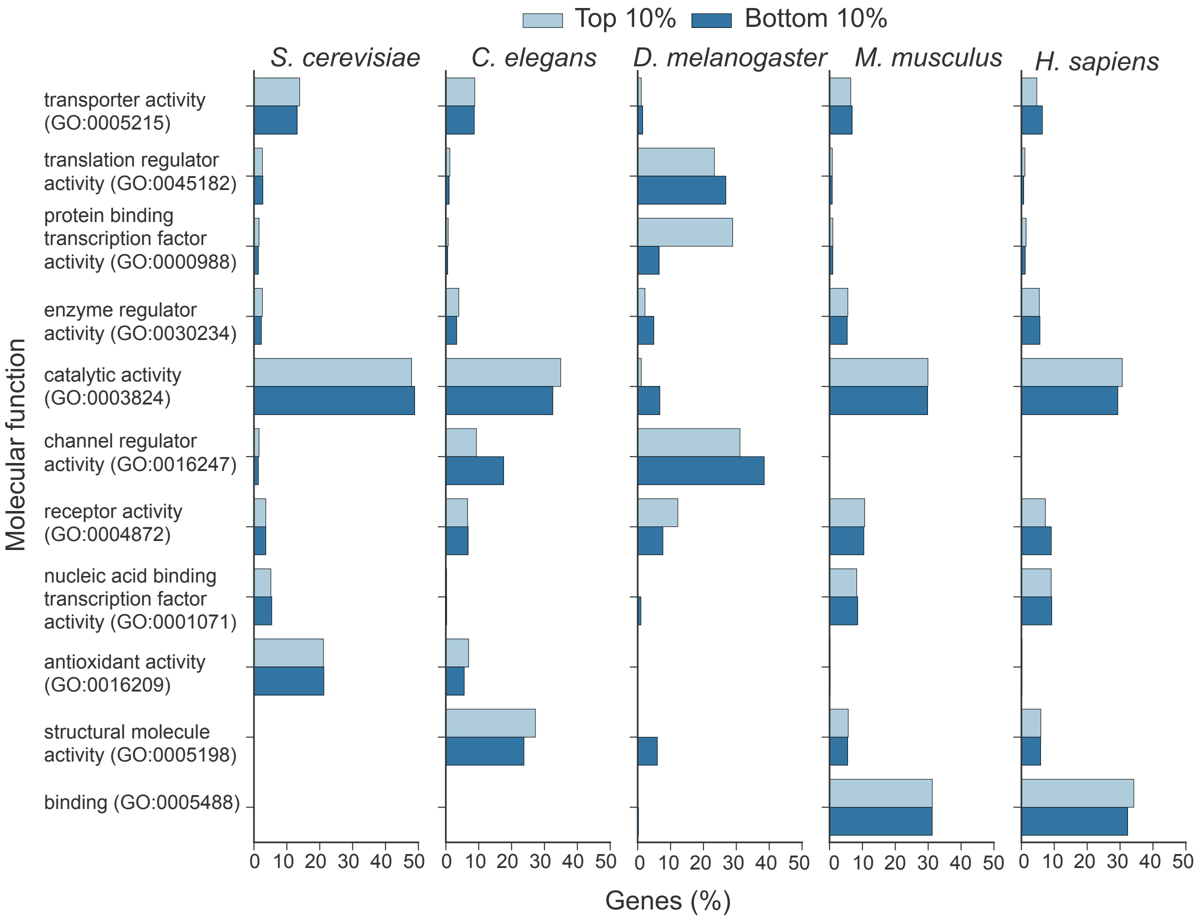

Figure S3 Representation of gene ontology annotations remains unbiased in correlated gene pairs. The molecular function classification of top and bottom 10% correlated gene pairs with intergenic distance below d_{exp} is shown for each organism.

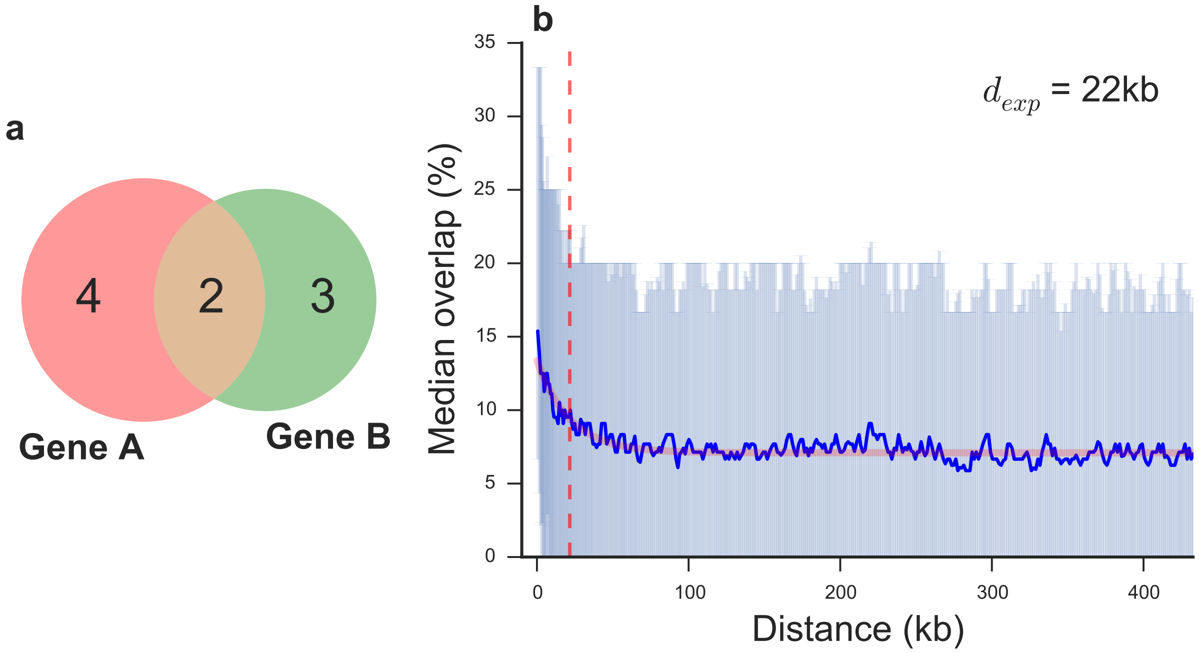

Figure S4 Gene pairs are correlated in spatial expression in *D. melanogaster*. The size of the intersection between the set of tissues in which each gene of a given pair is expressed was divided over the size of the union of the same sets. An example is shown in (a), where the percentage overlap is 2/(4+3-2)=0.4. b) Sliding median of the percentage overlap in tissue specific expression (blue line) and interquartile range (pale blue) with increasing intergenic distance. Fit to exponential decay function (red line) and corresponding d_{exp} (red dashed line) are shown.

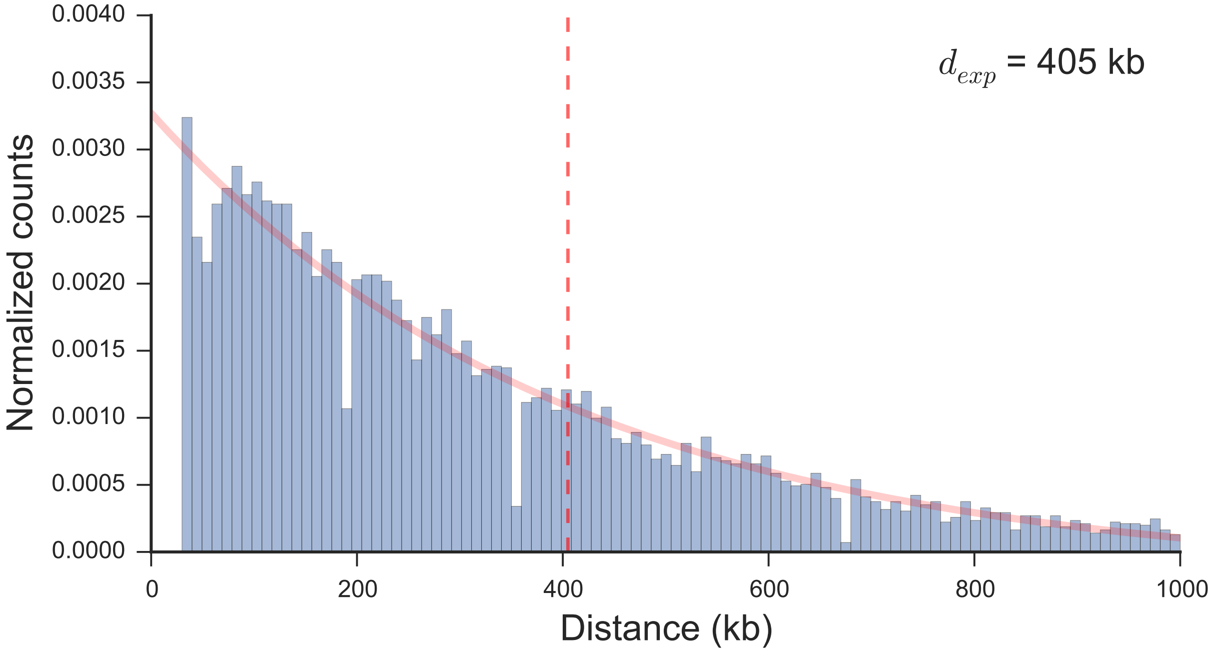

Figure S5 Chromatin looping decreases exponentially with distance in human cell lines. Normalized count of loops identified through HiC by Rao *et al. 2014* were fit to exponential decay function (red line); the resulting d_{exp} (red dashed line) is shown.

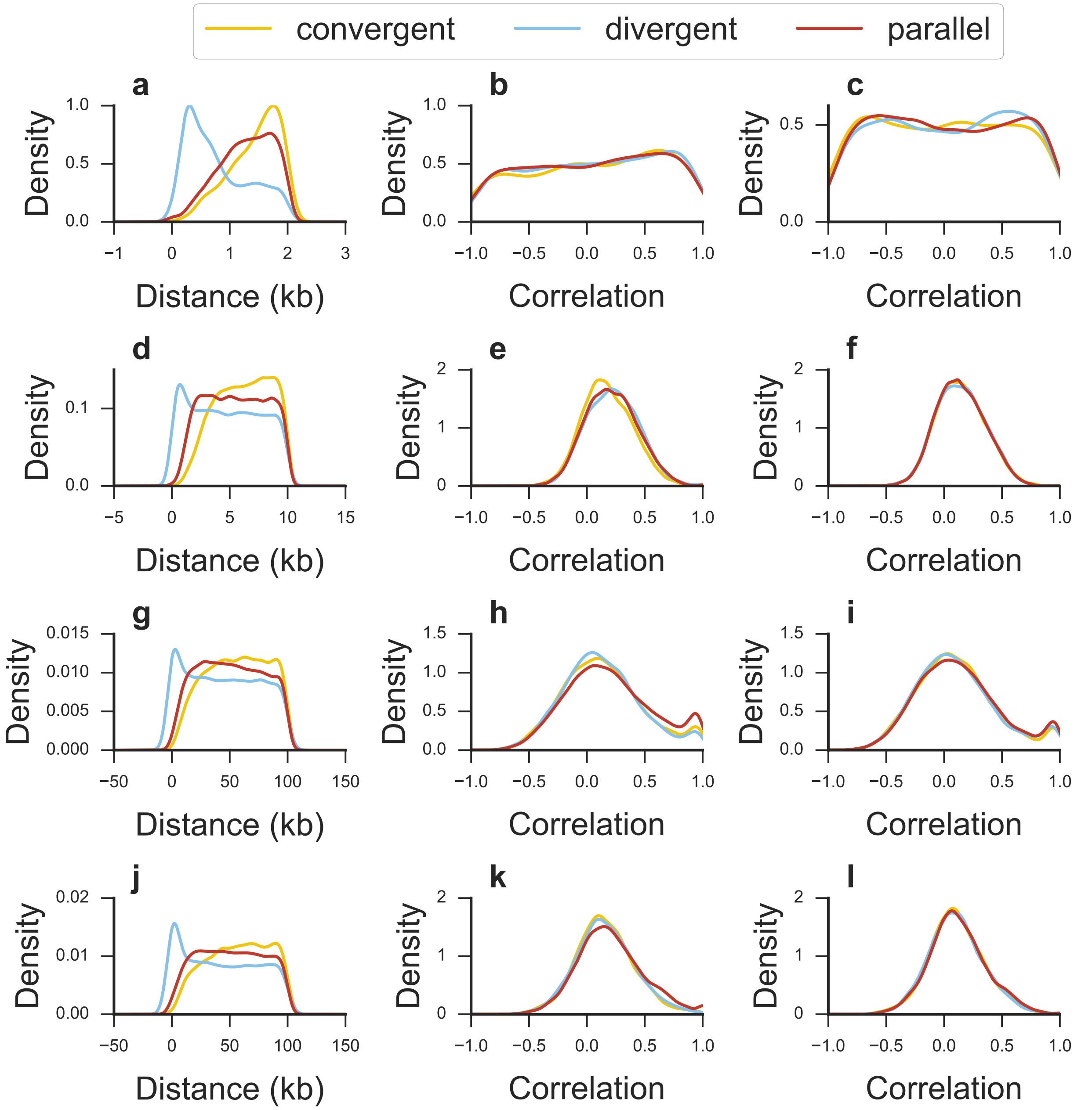

Figure S6 Gene orientation effect in correlation of gene pairs is explained by EP distance. Distribution of intergenic distances and the corresponding distribution of correlations of gene pairs is shown in the first and second columns, respectively; correlations after controlling for intergenic distance are shown in the third column. The range of distances between paired genes for each plot is as follows: *S. cerevisiae* below 2 kb (a,b) and between 2 and 4 kb (c). *C. elegans* below 10 kb (d,e) and between 10 and 20 kb (f). *H. sapiens* and *M. musculus* below 100 kb (g, h, j, k) and between 100 and 200 kb (i, l).
