# Supplementary figures and images for: Enhancer Sharing Promotes Neighborhoods of Transcriptional Regulation Across Eukaryotes
Source: G3 (Bethesda). 2016 Oct 31;6(12):4167–74. doi: 10.1534/g3.116.036228 (PMC5144984; doi:10.1534/g3.116.036228)

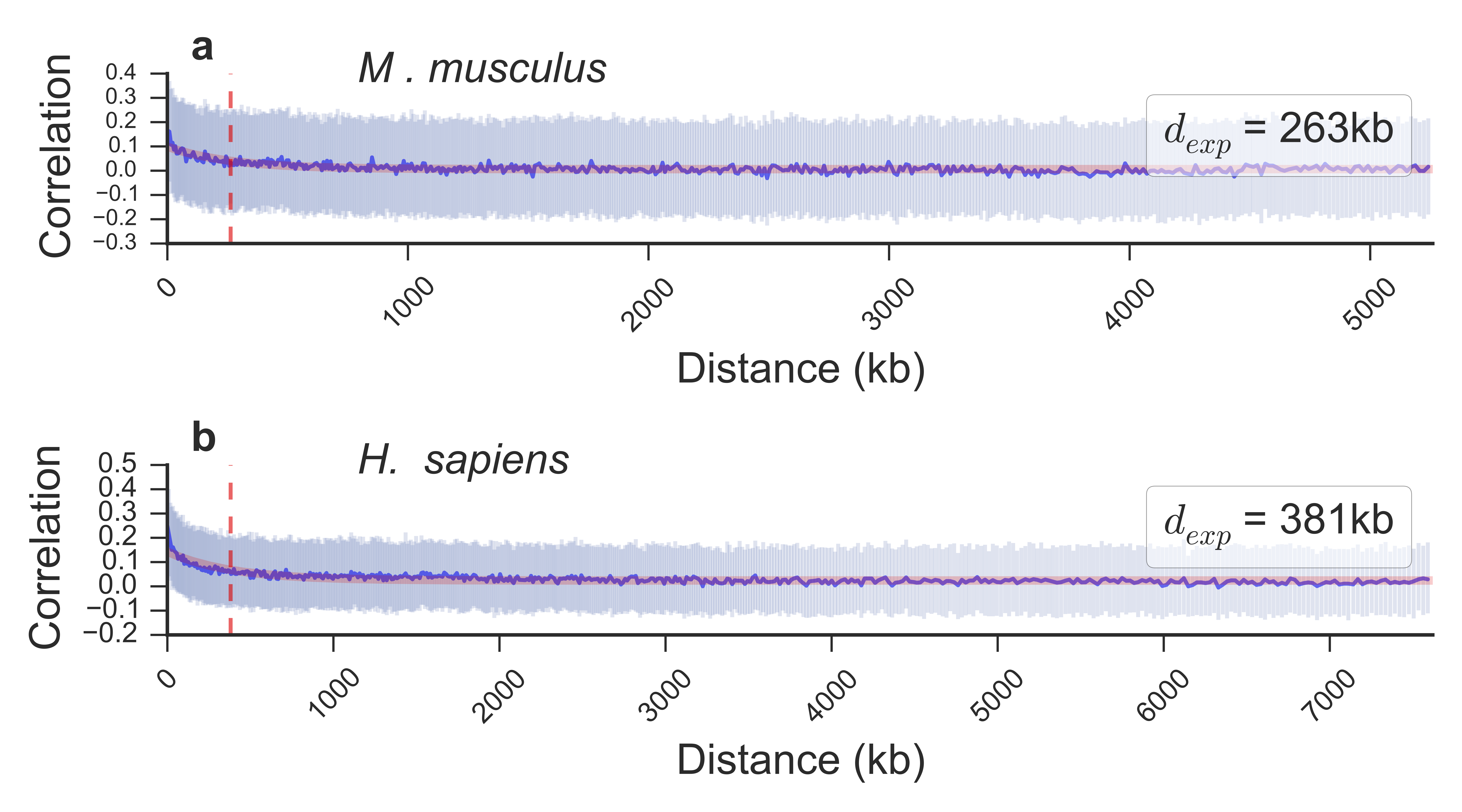

Supplement: Supplemental Material [file supp_g3.116.036228_FigureS2.jpg]

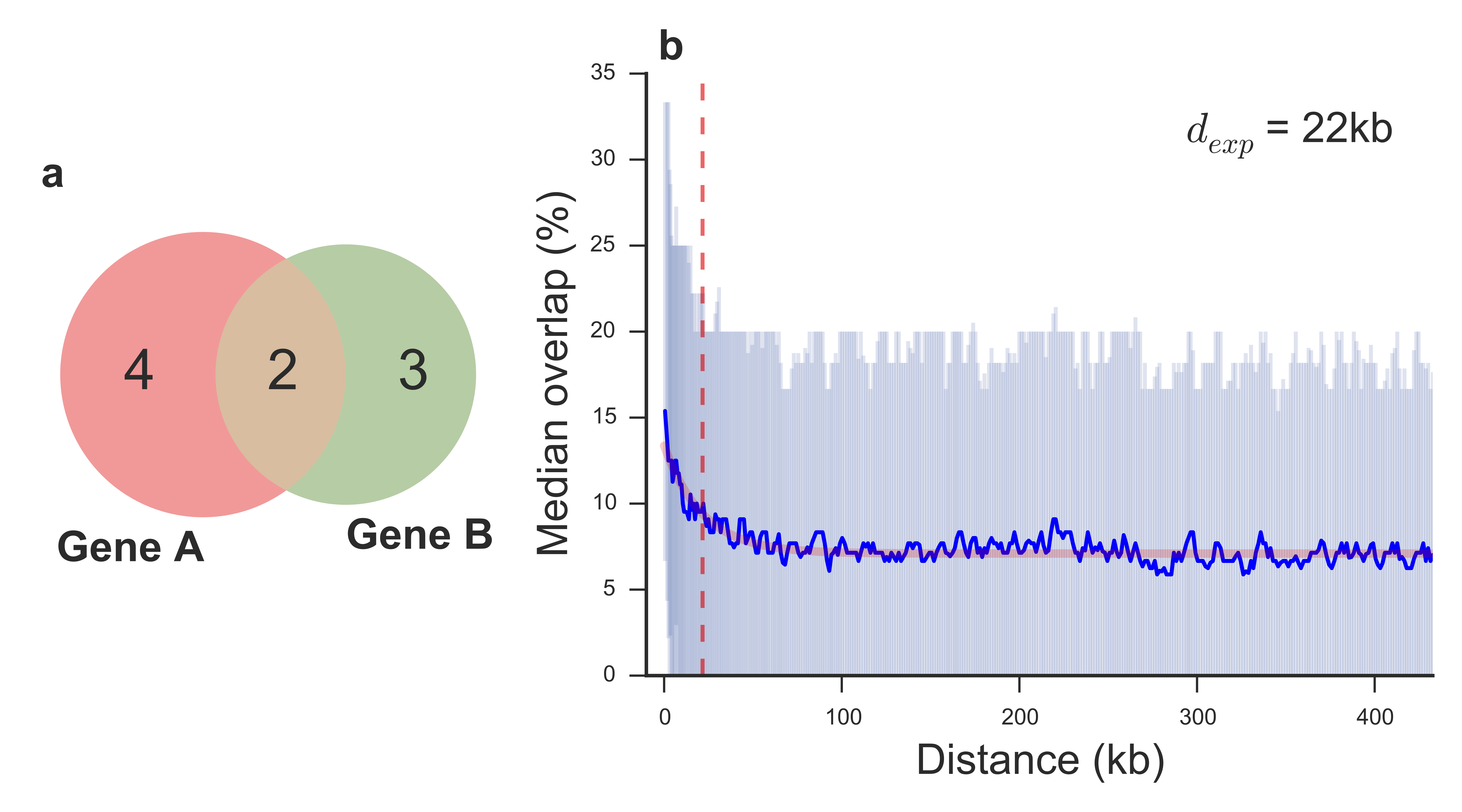

Supplement: Supplemental Material [file supp_g3.116.036228_FigureS4.jpg]

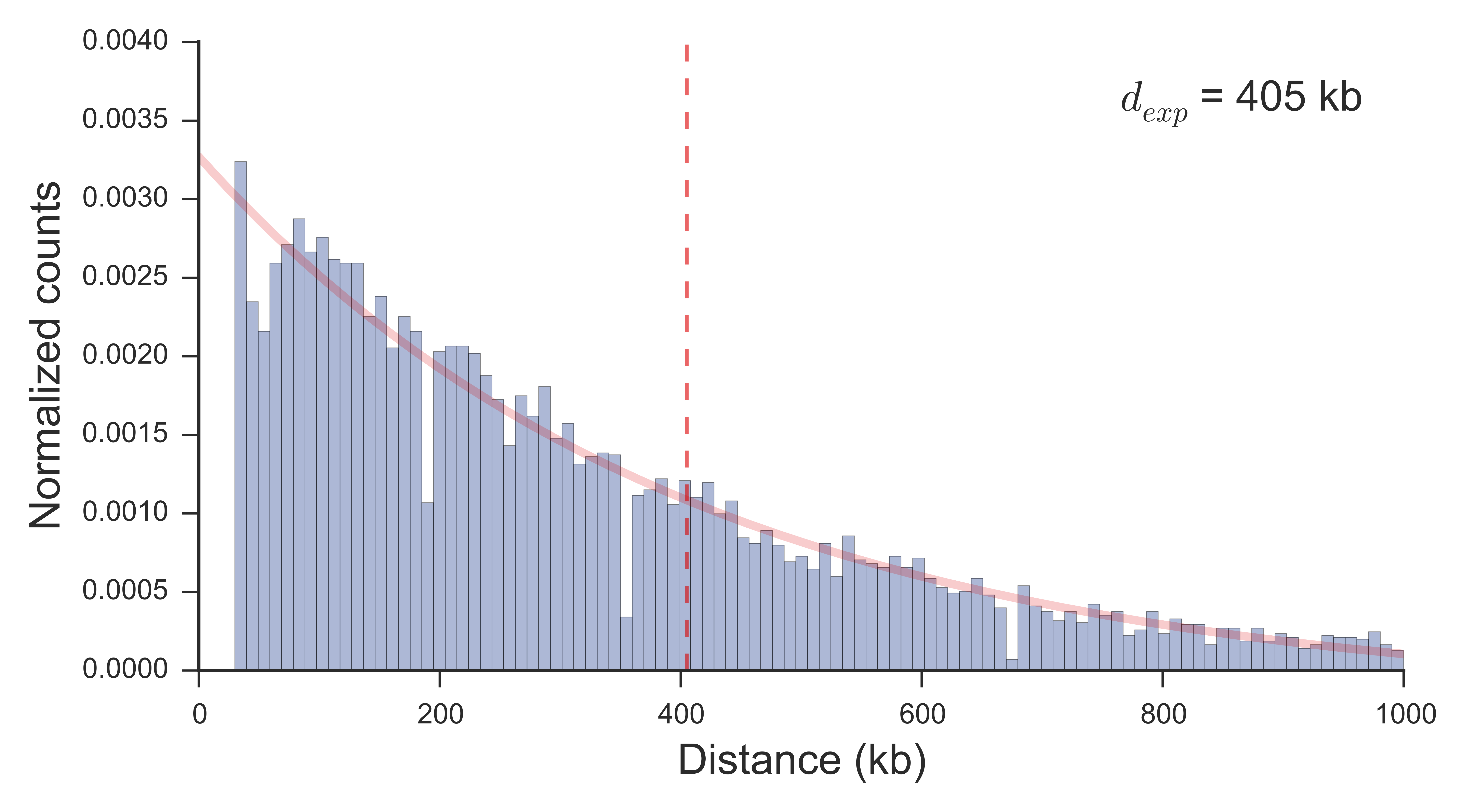

Supplement: Supplemental Material [file supp_g3.116.036228_FigureS5.jpg]
